# Parental Burnout in Israeli Parents of Children with ASD During Wartime: The Role of Child Behavior, Parental Emotion Regulation, Stress, and Social Support

**DOI:** 10.1007/s10803-024-06653-3

**Published:** 2024-12-23

**Authors:** Shani Aviad, Shlomit Shnitzer-Meirovich, Ayelet Gur

**Affiliations:** 1 The Academic College Levinski-Wingate, Tel-Aviv, Israel; 2 Social Work Department, Faculty of Social Sciences & Humanities, Tel-Hai College, Qiryat Shemona, Israel

**Keywords:** Parental burnout, Emotion regulation, Behavioral problems, Social support, ASD, War

## Abstract

During emergencies like wartime, parents of children with Autism Spectrum Disorder (ASD) face heightened challenges, potentially leading to Parental Burnout (PB). Wartime conditions can exacerbate children’s behavioral difficulties, contributing to PB. Protective factors such as successful Emotional Regulation (ER) and perceived social support may mitigate PB. This study aims to compare child behavioral problems, parental ER difficulties, perceived social support, stress, and PB between parents of children with ASD and Typical Development (TD) during wartime. It also investigates how ER difficulties, perceived social support, and stress moderate the relationship between child behavioral problems and PB. The study included 213 Israeli parents: 101 parents of children diagnosed with ASD and 112 parents of TD children. Participants were recruited during the “Swords of Iron” War using purposive sampling via online platforms. Findings indicate that children with ASD exhibited higher levels of behavioral problems, and their parents experienced increased difficulties in ER, stress, and PB, alongside decreased perceived social support compared to parents of TD children. Moderation analysis revealed that ER difficulties, perceived social support, and stress moderated the direct association between child behavioral problems and PB specifically among parents of TD children. This study underscores the heightened vulnerability of parents with ASD-diagnosed children during wartime, emphasizing the importance of understanding how these circumstances impact parental well-being and available resources. Effective interventions should target enhancing ER, social support, and addressing parental stress, PB, and child behavioral issues, underscoring the need for prioritized services despite wartime challenges.

## Introduction

War impacts every child by exposing them to danger, instilling fear, causing them to lose loved ones, forcing displacement, and disrupting their lives and education. These effects are even more profound for children with Autism Spectrum Disorder (ASD), who face additional challenges in accessing essential services, therapies, and educational opportunities (Ćerimovic, 2023). These challenging conditions may exacerbate their symptoms, increase behavioral and emotional difficulties, and reduce their mental well-being (White et al., 2021). Parents of children with ASD already face significant challenges and are at a higher risk of experiencing Parental Burnout (PB) compared to parents of children with Typically Development (TD; Kaba et al., 2023; Kutuk et al., 2021; Yılmaz et al., 2021). Therefore, it’s crucial to explore both personal and external support resources to help them cope effectively during such extraordinary times. The present study seeks to address this knowledge gap by investigating this issue among Israeli parents of children diagnosed with ASD during the “Swords of Iron” war.

### Parenting Children with ASD During Routine and Wartime

ASD is a neurodevelopmental disorder that typically emerges in early childhood, characterized by persistent challenges in two main areas: (1) social communication and interaction, and (2) restricted and repetitive behaviors (American Psychological Association, 2022). Alongside these primary symptoms, children with ASD often display emotional and behavioral difficulties such as hyperactivity, aggression, tantrums, refusal to engage in daily activities, disruptive behavior, and self-harming actions (Kaba et al., 2023; Yılmaz et al., 2021). As of March 2022, approximately 1 in 100 children were diagnosed with ASD (World Health Organization, 2022), with the prevalence of ASD continuing to rise (Salari et al., 2022).

Parents of children with ASD face high demands, from navigating the diagnosis process to coordinating various aspects of their child’s care and education. This increased responsibility can lead to emotional and physical strain, potentially resulting in fatigue, anxiety, and depression (Ardic, 2020; Ardic & Olcay, 2021; Kocak et al., 2023; Lin et al., 2023), which may impede the ability to parent effectively and disrupt family relationships (Ardic, 2020; Kocak et al., 2023; Shepherd et al., 2024). Furthermore, these parents may experience social support deficits and increased feelings of loneliness due to fear of stigma (Kocak et al., 2023). Research has shown that families raising a child with ASD face unique challenges that can affect their overall well-being. Not only do individual parents experience increased mental health risks, but the entire family unit may also experience a diminished quality of life compared to families with TD children. This finding emphasizes the importance of considering the broader impact of reduced parental well-being on the parent-child relationship. The potential deterioration of parent-child attachments resulting from these challenges may, in turn, negatively influence the child’s development and overall quality of life (Shepherd et al., 2024).

During emergencies, parents of children with ASD often face additional challenges compared to the general population. The disruption of routine poses significant difficulties for these children due to their strict adherence to schedules and struggles with changes (Amorim et al., 2020; Bentenuto et al., 2021; Manning et al., 2021; Mutluer et al., 2020; Türkoğlu et al., 2021; White et al., 2021; Yilmaz et al., 2021). Furthermore, reduced physical and social interaction, along with disruptions in special education and treatments, can severely impact the maintenance and progress of their functioning (Çi̇men et al., 2023; Kaba et al., 2023; White et al., 2021). Studies have found that parents’ difficulty in dealing with their child’s additional behavioral problems is strongly correlated with their stress level, often more so than the child’s typical ASD symptoms (Ardic & Olcay, 2021; Argumedes et al., 2018; Shepherd et al., 2018). The ASD literature has documented the transactional effects arising from the reciprocal relationship between parenting stress and the frequency and impact of child externalizing behaviors (Shepherd et al., 2024). The responsibility added to parents during disruptions in children’s education and care settings, combined with a decrease in social support and an increase in the child’s challenging behavior, adds to the emotional, psychosocial, and mental burden of already stressed parents, increasing the risk of PB (Alhuzimi, 2021; Kaba et al., 2023; Yılmaz et al., 2021).

### PB among Parents of Children with ASD

PB, as defined by Burisch (2006), refers to a chronic stress-related syndrome experienced within the parental role, characterized by persistent physical and mental fatigue. It represents prolonged distress resulting from a lack of parental resources to manage parenting stress over a minimum period of three months. PB encompasses four primary dimensions: (1) enduring physical and emotional exhaustion within the parental role (chronic fatigue); (2) emotional detachment from the child, with parents focusing solely on meeting the child’s basic needs; (3) feeling overwhelmed by the parental role, leading to a sense of saturation and an inability to find fulfillment in parenting; and (4) a notable contrast between present parenthood and past experiences of parenthood. PB carries various detrimental effects on the mental and physical well-being of the parent, on marital relationships, and on the children involved (Ardic, 2020; Ardic & Olcay, 2021; Lin et al., 2022; Lin et al., 2023; Mikolajczak & Roskam, 2018; Mikolajczak et al., 2019; Yang et al., 2021).

Previous studies have indicated that parents of children with ASD are likely to experience more PB. As mentioned, they are compelled to contend with chronic stress stemming from developmental differences and escalating childcare demands, which adds to the burden and fatigue experienced by parents of TD children, and even surpasses that of parents of children with other disabilities (Ardic & Olcay, 2021; Çi̇men et al., 2023; Kutuk et al., 2021; Lin et al., 2023). Additional factors contributing to heightened PB may include challenges in accessing services, limited participation in social activities, and financial strain (Ardic, 2020).

To the best of our knowledge, there have been no studies specifically examining PB among parents of children with ASD during times of war. However, research conducted during other emergencies, such as the COVID-19 pandemic, suggests that these parents are at an increased risk for PB during wartime. This is due to the unprecedented and significant demands they face during this time, including burdens related to remote schooling and work, increased caregiving responsibilities, and heightened symptoms of depression and anxiety (Findling et al., 2023; Holly et al., 2024; Yılmaz et al., 2021). In addition to these similarities between times of war and the COVID-19 pandemic, the current situation in Israel since 10/7/23 (1st day of the “Swords of Iron” war) is differentiated in the gravity of the immediate threat to all community members and the ongoing changing threat to the state of Israel. Hence, it is crucial during this extreme wartime to pinpoint the resources or protective factors that can assist parents in managing the heavy load of wartime challenges. In this study, we investigated personal factors such as ER and stress, as well as the external factor of perceived social support, which may have a positive impact on parents’ overall psychological well-being and specifically on PB (Mikolajczak & Roskam, 2018).

### Social Support Among Parents of Children with ASD

Perceived social support, which encompasses various forms of assistance from one’s social network, plays a vital role in predicting overall psychological well-being and mitigating parental stress, depression, and burnout (Ardic, 2020; Hsiao, 2016). It serves as a significant protective factor against PB, surpassing even personality traits and socio-demographic factors (Ardic, 2020; Kocak et al., 2023; Lin et al., 2022; Lin et al., 2023; Yamoah & Brown, 2023). Not only does social support offer practical aid such as information and financial assistance, but it also fosters parental resilience in the face of challenges (Lin et al., 2022, 2023).

The experience of parenting a child with ASD emphasizes the importance of social support in addressing PB at different stages of the parenting journey (Yamoah & Brown, 2023). Social support has been identified as a critical factor in reducing the negative psychological effects of raising a child with ASD and enhancing the perception of the quality of life for parents of children with ASD (Kuru & Piyal, 2018; Luther et al., 2005). Additionally, the support received by parents of children with ASD through therapeutic services, face-to-face or online support groups, close relationships, financial assistance, and support from authorities has been found to significantly influence their level of PB (Kocak et al., 2023).

Although research on the social support of parents of children with ASD during wartime is limited, a systematic review on parents of children with ASD during COVID-19 indicates the importance of social support in helping them cope with the negative effects of the extraordinary situation marked by uncertainty, disasters, and unexpected changes. All types of social support were found to facilitate the caregiving burden of parents of children with ASD (Yılmaz et al., 2021). For this reason, during such emergency times, it is crucial for parents of children with ASD to maintain contact with all available sources of support, such as other parents, teachers, therapists, healthcare professionals, community resources, and authorities (Yılmaz et al., 2021).

Social support is not always available, especially during emergencies such as war, when the regular functioning of social systems, interpersonal relationships, and outdoor activities are disrupted. However, the absence of social support does not necessarily result in PB for all parents, as it can be compensated for by internal resources such as high ER skills (Lin et al., 2022; Mikolajczak & Roskam, 2018).

### ER Difficulties of Parents of Children with ASD

ER involves individuals’ ability to control the emotions they experience, the timing of these emotions, and how they express them (Gross, 1998). Effective ER entails several components: (a) evaluating contextual demands, (b) possessing a range of strategies to choose from, and (c) monitoring and adjusting strategies based on their effectiveness (Bonanno & Burton, 2013; Brandão et al., 2024). Unlike inherent personality traits, ER strategies are skills that can be taught and learned through therapy, leading to meaningful changes in emotional experiences and symptoms reduction (Hallion et al., 2018; Wang et al., 2021). Adaptive ER strategies are often associated with positive outcomes and are regarded as protective factors against the development of psychopathology (Aldao et al., 2010; Lincoln et al., 2022). Conversely, maladaptive ER strategies are considered risk factors for psychopathology development and are linked to negative outcomes such as diminished well-being and impaired interpersonal relationships (McRae & Gross, 2020).

The development of ER skills among parents of children with ASD holds utmost importance due to the significant challenges posed by the behavioral and emotional issues of these children. Compared to parents of TD children, parents of children with ASD are exposed to higher levels of emotionally related risk factors (Kerr et al., 2021; Lin et al., 2021; Prikhidko & Swank, 2019). Research suggests that proficient ER skills can mitigate the risk of PB among these parents, compensating for the lack of social support and heightened stress (Brandão et al., 2024; Lin et al., 2022; Prikhidko & Swank, 2019; Rodriguez et al., 2020). Additionally, employing effective ER strategies by parents has been associated with reduced negative effects of PB on their children’s mental health (Yang et al., 2021). It appears that adept ER strategies aid parents in coping with the demands of parenting and moderating their adverse effects, thereby mitigating the impact of risk factors related to children’s variables (e.g., difficult temperament, disability, behavioral problems) on PB (Lin et al., 2022; Swit & Breen, 2023).

Examining these relationships during emergency times holds additional significance. Although research on the ER of parents of children with ASD during wartime is limited, investigations into the role of ER on PB during the Covid-19 pandemic have shown that ER is strongly associated with PB and serves as a significant moderator of the association between the negative effects of Covid-19 and PB (Prikhidko et al., 2020; Santelices et al., 2022; Swit & Breen, 2023; Vertsberger et al., 2022).

### Study Context and Objective

The current study was conducted during the “Swords of Iron” conflict between Israel and Hamas. On October 7, 2023, about 6000 terrorists of the Hamas terrorist organization infiltrated Israeli territory and civilian communities. Approximately 1,200 Israeli citizens and foreign residents were murdered in the attack, and about 240 people were taken hostage. Simultaneously, the attack included artillery fire of 4,300 rockets and a number of mortar bombs on Israel. As the outbreak of war ensued, Israel evacuated approximately 126,000 evacuees, and additional tens of thousands of residents decided to evacuate independently due to a sense of security threat and an inability to maintain a reasonable lifestyle (Shahar & Lerer, 2024). Consequently, Israel’s welfare system was presented with unprecedented challenges and the entire education system was shutdown for several weeks (Taub Center researchers, 2023). These conditions accentuate the necessity to examine the role of parents’ ER, stress, and social support in the association between a child’s behavioral problems and PB particularly during such an emergency situation. The goal of the current study is to examine these association among parents of children with ASD and compare them to parents of children with TD. Since there is a lack of research addressing PB among parents of children with ASD during wartime, the current study poses research questions instead of hypotheses:

1. Do parents of children with ASD differ from parents of children with TD in terms of the child’s behavioral problems, parental ER difficulties, stress, perceived social support, and levels of PB during wartime, as previous studies have found during routine circumstances?
2. Are the child’s behavioral problems, parental ER difficulties, stress, and perceived social support correlated with PB during wartime among both parents of children with ASD and TD, as previous studies have shown these correlations during routine circumstances?
3. Do parental ER difficulties, stress, and perceived social support serve as moderation variables in the association between the child’s behavioral problems and PB during wartime among both parents of children with ASD and TD?

## Method

### Participants

The current study comprised of 213 parents (34 males and 179 females) aged 29 to 61 (*M* = 44.00, *SD* = 5.90). 101 of the parents were parents of children with ASD (16 males and 85 females) and 112 of the parents were parents of children with TD (18 males and 94 females). The parents were asked about the number of children they have at home, with responses ranging from 1 to 5 children (*M* = 2.52, *SD* = 0.85). A t-test revealed no significant difference between the two parent groups in the number of children at home, *t*(211) = 1.23, *p* =.219 (ASD group: *M* = 2.59, *SD* = 0.79; TD group: *M* = 2.45, *SD* = 0.91).

The ASD diagnosis of all children was made by a clinical psychologist, developmental physician, neurologist, or child psychiatrist. The parents were administered with short background questionnaire (see Table 1).

**Table 1:** Parent’s characteristics by group (*N* = 213)

Characteristics		Group			
	Values	Parents of children with ASD (*n* = 101)	Parents of children with TD (*n* = 112)	*X* ^2^	*p*
***Demographic characteristics***					
Parent’s gender	Male	16 (15.8%)	18 (16.1%)		
	Female	85 (84.2%)	94 (83.9%)	0.00	0.964
Education^1^	High school	12 (11.9%)	4 (3.6%)		
	Professional certificate	13 (12.9%)	2 (1.8%)		
	B.A.	38 (37.6%)	39 (35.1%)		
	M.A.	31 (30.7%)	58 (52.3%)		
	PhD.	7 (6.9%)	8 (7.2%)	4101.00	< 0.001
Marital status	Married/shared parenting	81 (80.2%)	97 (86.6%)	1.59	0.208
	Divorced/separated	10 (9.9%)	8 (7.1%)	0.52	0.470
	Single parent	10 (9.9%)	7 (6.3%)	0.96	0.326
***Changes or implications of the war***					
Change in work following the war	No	93 (92.1%)	108 (96.4%)		
	Yes	8 (7.9%)	4 (3.6%)	1.89	0.169
Change in residence following the war	No	68 (67.3%)	80 (71.4%)		
	Yes	33 (32.7%)	32 (28.6%)	0.42	0.516
A change in the main caregiver of the child(ren) following the war	No	89 (88.1%)	103 (92.0%)		
	Yes	12 (11.9%)	9 (8.0%)	0.88	0.347
Change in the educational framework of the child(ren) following the war	No	53 (52.5%)	72 (64.3%)		
	Yes	48 (47.5%)	40 (35.7%)	3.05	0.080
Change in supports, treatments, or routine classes of the child(ren).	No	50 (49.5%)	67 (59.8%)		
	Yes	51 (50.5%)	45 (40.2%)	2.28	0.131
A family member or close acquaintance who was injured/killed in the war	No	88 (87.1%)	102 (91.1%)		
	Yes	13 (12.9%)	10 (8.9%)	0.86	0.355
A family member or close acquaintance who serves in the army	No	52 (51.5%)	46 (41.1%)		
	Yes	49 (48.5%)	66 (58.9%)	2.32	0.128

*p <.05, **p <.01, ***p <.001; ^1^Variable in an ordinal scale – Mann-Whitney was conducted

As Table 1 shows, Mann-Whitney analysis indicated that the parents in the TD group were at a higher level of education compared to the parents in the ASD group. Chi-square analysis indicated that there were no statistically significant differences between the two groups in all other background characteristics nor in changes caused by the war.

### Materials

#### Background Questions

The parents of children with ASD were asked about their gender, education, and marital status. Additionally, they were questioned about several changes in routine during the ‘Swords of Iron’ war: whether they experienced changes in work or residence, whether there was a change in the main caregiver of their children, and whether their children’s education and therapies were disrupted. Moreover, they were asked if they had a family member or close acquaintance who was injured or killed in the war, and if they had a family member or close acquaintance who served in the army during the war.

#### The Aberrant Behavior Checklist (ABC)

Developed by Aman et al. (1985), is a standardized tool used to assess problem behaviors in individuals, with caregivers rating 58 items based on severity using a 4-point Likert scale (ranging from 0 for “not at all a problem” to 3 for “severe problem”). Originally, the ABC includes five sub-scales: irritability, social withdrawal, stereotypy, hyperactivity, and inappropriate speech. For our study, we focused on the 15-item irritability sub-scale and the 16-item hyperactivity/noncompliance sub-scale. A comprehensive analysis by Kaat et al. (2014) involving 1,893 youth with ASD found that the irritability sub-scale of the ABC explains the most variation in parent-reported problem behaviors. Consistent with prior findings (Aman et al., 1985), the highest correlations were observed between the irritability and hyperactivity/noncompliance sub-scales. The internal consistency, as measured by Cronbach’s alpha, was high for both the overall 31-item scale (α = 0.97) and specifically for the irritability (α = 0.94) and hyperactivity/noncompliance (α = 0.96) sub-scales.

#### The Parental Burnout Assessment (PBA)

Developed by Roskam et al. (2018), consists of 23 items used to measure PB. The questionnaire was translated into Hebrew using the translation–back-translation method (Findling et al., 2023). It assesses four dimensions of PB: physical and emotional exhaustion in the parental role (9 items), emotional distancing from the child (3 items), saturation from the parental role (5 items), and contrast with the previous parental self (6 items). Parents were asked to rate the frequency of these emotions experienced since October 7th (the first day of the war) on a scale from 1 (“not at all”) to 7 (“every day”). The internal consistency, as measured by Cronbach’s alpha, was high for all 23 items (α = 0.97) and for each of the four dimensions individually (α = 0.94, α = 0.85, α = 0.91, and α = 0.93, respectively).

#### The Difficulties in Emotion Regulation Scale (DERS)

Developed by Gratz and Roemer (2004), is widely recognized as a comprehensive tool for assessing difficulties in ER. It aims to evaluate various aspects of emotion dysregulation across cognitive, affective, and behavioral domains (Sörman et al., 2022). The scale comprises 36 items divided into six subscales. Each item is rated on a 5-point Likert scale, ranging from 1 (almost never) to 5 (almost always), where higher scores indicate greater difficulties in ER, with total scores ranging from 36 to 180. The six subscales of the DERS are: (1) Nonacceptance (6 items, reflecting nonacceptance of emotional responses). (2) Goals (5 items, reflecting difficulties in pursuing goal-directed behavior during stress). (3) Impulse (6 items, reflecting impaired control over impulsive behaviors when distressed). (4) Awareness (6 items, reflecting lack of emotional awareness). (5) Strategies (8 items, reflecting limited access to effective ER strategies). (6) Clarity (5 items, reflecting lack of emotional clarity). The internal consistency, assessed using Cronbach’s alpha, was high for all 36 items (α = 0.95) and for each of the six subscales individually (α = 0.91, α = 0.86, α = 0.89, α = 0.80, α = 0.90, and α = 0.77, respectively).

#### The Multidimensional Scale of Perceived Social Support (MSPSS)

Developed by Zimmet et al., (1988), is a self-report tool designed to assess individuals’ subjective perceptions of the social support available to them from three different sources: family, friends, and significant others. It consists of 12 items, with each source of support represented by 4 items. Participants were asked to rate the extent to which each statement reflected their feelings since October 7th on a 7-point Likert scale, where 1 indicates “not at all suitable” and 7 indicates “very suitable”. The perceived social support score was calculated by averaging the scores of items within each source, resulting in scores ranging from 1 to 7. Higher scores indicate greater perceived social and family support. The internal consistency, as measured by Cronbach’s alpha, was high for all 12 items combined (α = 0.94) and also for each of the three sources of support individually (α = 0.94 for family support, α = 0.94 for friend support, and α = 0.93 for significant other support).

#### Perceived Stress Scale

Stress levels were assessed using a self-report questionnaire developed by Cohen et al. in 1983. This questionnaire examines how individuals perceive and experience stress in their lives over a recent period. It consists of 14 items and responses were recorded on a 4-point Likert scale, where 1 indicates “never” and 4 indicates “often”. The overall score for the questionnaire is derived from averaging the scores of all 14 items. A higher score indicates higher perceived stress levels, whereas a lower score indicates lower stress levels. The internal consistency, assessed using Cronbach’s alpha, was high for all 14 items (α = 0.91).

### Procedure

After receiving ethics approval, the data were collected using an online questionnaire constructed via the Qualtrics software. The sampling method of the parents was purposive sampling. In this sampling method, the subjects are purposefully selected based on the characteristics suitable for the study (Etikan et al., 2015). The questionnaire was administered via social networks and forums of parents of children with ASD. The parents who participated in the study signed an informed consent form. It was emphasized to the parents that the questionnaire is anonymous, and that they have the option to stop their participation in the study at any time.

### Data Analyses

The first goal of the current study was to examine the differences in the study measures between the two parent groups. To examine these differences, t-test analyses were conducted for each measure. Pearson correlation analyses were conducted among the entire sample and each group to examine the second goal regarding the association between the child’s behavioral problems, parents’ ER difficulties, perceived social support, parental stress, and PB during the “Swords of Iron” war. To examine the contribution of parent’s and children’s background characteristics as well as the changes in the family routine to the PB during the “Swords of Iron” war, multiple regression analyses were conducted for each group. Finally, to examine the moderation of the parent ER difficulties, social support and perceived stress in the association between child behavioral problems and PB during the “Swords of Iron” war, moderation analysis using model 1 was conducted using the PROCESS software (Hayes, 2018).

## Results

### Differences Between Parents of Children with ASD and Parents of Children with TD During Wartime in Children’s Behavioral Problems, Parents’ ER Difficulties, Perceived Social Support, Stress, and PB

The first goal of the current study was to examine the differences in the study measures between the two parent groups. To examine these differences, t-test analyses were conducted for each measure. Significant differences were found in the child’s behavioral problems reported by the parents (total scale and the two sub-scales), parents’ ER (total scale and four out of six sub-scales), stress, and PB (total score and the four sub-scales), indicating higher scores among parents of children with ASD. On the contrary, parents perceived social support (total scale and two out of three support resources) was significantly higher among parents of children with TD. No significant differences between the two groups were found in the two subscales of DERS: Lack of emotional awareness and Lack of emotional clarity, and in the level of support from significant others (See Table 2).

**Table 2:** Mean, SD and t-values of the child’s behavioral problems, parents ER difficulties, parents’ perceived social support, parental stress and PB during “Swords of Iron” war according to group

Study measures	Group							
	Parents of children with ASD (*n* = 101)			Parents of children with TD (*n* = 112)		t-values		
	M	SD		M	SD	t	***p***	d^#^
**Child behavioral problems (Scale 0–3)**								
Total scale	1.03	0.74		0.43	0.49	6.88***	< 0.001	0.96
Irritability	0.96	0.75		0.42	0.49	6.23***	< 0.001	0.87
Hyperactivity/ Noncompliance	1.08	0.80		0.44	0.52	6.91***	< 0.001	0.97
**Parent ER difficulties (Scale 1–5)**								
Total scale	2.53	0.74		2.10	0.57	4.66***	< 0.001	0.65
Nonacceptance of emotional responses	2.72	1.13		2.07	0.88	4.65***	< 0.001	0.65
Difficulty engaging in goal-directed behavior	2.84	1.07		2.39	0.75	3.53***	< 0.001	0.49
Impulse control difficulties	2.46	1.14		1.84	0.63	4.86***	< 0.001	0.69
Lack of emotional awareness	2.67	0.85		2.59	0.73	0.77	0.441	0.11
Limited access to emotion regulation strategies	2.50	0.96		1.96	0.71	4.64***	< 0.001	0.65
Lack of emotional clarity	1.97	0.73		1.84	0.63	1.36	0.177	0.19
**Social Support (Scale 1–7)**								
Total scale	4.83	1.45		5.51	1.41	3.43***	< 0.001	0.47
Family support	4.51	1.92		5.42	1.62	3.72***	< 0.001	0.51
Friend support	4.55	1.89		5.32	1.59	3.20**	0.002	0.44
Significant other support	5.44	1.54		5.78	1.49	1.64	0.103	0.22
**Perceived Stress (Scale 1–4)**								
Stress	2.66	0.65		2.37	0.51	3.57***	< 0.001	0.50
**PB (Scale 1–6)**								
Total scale	2.27	1.17		1.65	0.79	4.44***	< 0.001	0.63
Exhaustion in parental role	2.69	1.27		1.93	0.95	4.88***	< 0.001	0.68
Emotional distancing	1.92	1.24		1.48	0.74	3.11**	0.002	0.44
Feelings of being fed up	1.83	1.11		1.34	0.69	3.78***	< 0.001	0.53
Contrast in parental self	2.17	1.32		1.58	0.89	3.78***	< 0.001	0.53

**p <.01, ***p <.001; ^#^d = Cohen’s d effect size

### The Association Between the Child’s Behavioral Problems, Parents’ ER Difficulties, Perceived Social Support, Stress, and PB During the “Swords of Iron” War

The second goal of the current study was to examine the association between the child’s behavioral problems, parents’ ER difficulties, perceived social support, stress, and PB during the “Swords of Iron” war among the entire sample and each group. Pearson correlation analyses indicated that the child’s behavioral problems, parents’ ER difficulties, and the stress scales were positively correlated with PB. All correlation coefficients were moderate to high (Cohen, 1987). Additionally, the results indicated that the child’s behavioral problems, parents’ ER difficulties, stress, and PB scales were negatively correlated with the degree of social support. All correlation coefficients were moderate. Finally, the correlations between social support and PB among parents of children with TD during the “Swords of Iron” war did not reach a significance level [*r*(99) = − 0.10, *p* =.306] (See Table 3).

**Table 3:** Pearson correlation coefficients between the child’s behavioral problems, parents ER difficulties, perceived social support and stress and PB during “Swords of Iron” war among all sample and each group

	2	3	4	5
**All sample (*N*** **= 213)**				
Child behavioral problems (1)	0.60***	− 0.38***	0.62***	0.59***
Parent ER difficulties (2)		− 0.47***	0.73***	0.45***
Social support (3)			− 0.43***	− 0.30***
Perceived stress (4)				0.56***
PB (5)				1
**Parents of children with ASD (*n*** **= 101)**				
Child behavioral problems (1)	0.59***	− 0.37***	0.67***	0.53***
Parent ER difficulties (2)		− 0.44***	0.78***	0.42***
Social support (3)			− 0.44***	− 0.33***
Perceived stress (4)				0.61***
PB (5)				1
**Parents of children with TD (*n*** **= 112)**				
Child behavioral problems (1)	0.50***	− 0.31***	0.47***	0.56***
Parent ER difficulties (2)		− 0.42***	0.60***	0.28**
Social support (3)			− 0.36***	− 0.10
Perceived stress (4)				0.38***
PB (5)				1

**p <.01, ***p <.001

Aside from correlation analyses, multiple regression analyses were carried out for each group to explain the variance in PB. These analyses aimed to examine whether there is a contribution of the parents’ characteristics and changes or implications of the war that the parents experienced during the “Sword of Iron” war to explaining the variation in the PB experienced by parents during the wartime.

The results of the regression analyses indicated that a change in residence following the war was the only variable that significantly explained the level of PB among parents of children with TD (*p* =.003). This contribution of this variable was marginally significant among parents of children with ASD (*p* =.056).

Regarding the parents of children with ASD, significant contribution to the EPV of the PB level was also found for the change in the educational settings, the change in the main caregiver, change in supports, treatments, or routine, and whether a family member or close relative was injured or killed following the war (See Table 4).

**Table 4:** Multiple regression results for the PB during “Swords of Iron” war in each group

Explanatory variable	Parents of children with ASD (*n* = 101)				Parents of children with TD (*n* = 112)		
	B	SE.B	β		B	SE.B	β
Parent’s gender	0.50	0.26	0.16		− 0.24	0.20	− 0.11
Education	− 0.04	0.09	− 0.04		− 0.09	0.10	− 0.09
Marital status	− 0.18	0.26	− 0.06		0.10	0.26	0.04
Parent’s age	− 0.01	0.02	− 0.03		0.00	0.01	0.00
Number of children	− 0.04	0.11	− 0.03		0.05	0.11	0.05
Change in work following the war	− 0.52	0.26	− 0.21*		0.18	0.17	0.10
Change in residence following the war	− 0.79	0.41	− 0.18		1.32	0.44	0.31**
A change in the main caregiver of the child(ren) following the war	1.31	0.35	0.37***		− 0.09	0.27	− 0.03
Change in the educational framework of the child(ren) following the war	0.70	0.22	0.30***		0.16	0.16	0.10
Change in supports, treatments, or routine classes of the child(ren).	0.94	0.24	0.40***		0.14	0.16	0.09
A family member or close acquaintance who was injured/killed in the war	− 0.79	0.29	− 0.23**		− 0.23	0.26	− 0.08
A family member or close acquaintance who serves in the army	0.39	0.21	0.17		0.08	0.15	0.05
**Explained Variance (EPV)**	**R**^**2**^ **= 44.3%**				**R**^**2**^ **= 23.8%**		

*p <.05, **p <.01, ***p <.001; note: In all explanatory variables 0 = No and 1 = Yes

### Parent ER, Perceived Social Support and Stress as Moderating Variables

The third goal of the study was to examine whether parent ER, perceived social support, and stress are moderating variables of the direct association between the degree of the child’s behavioral problems and the PB level during wartime. To examine this research question, moderation analyses using model 1 in PROCESS software (Hayes, 2018) were conducted. The background variables that were found to contribute to the EPV of the PB level in the regression analyses were taken as covariate variables in the moderation analyses. The findings revealed that parent ER, perceived social support, and stress are moderating variables between the degree of the child’s behavioral problems and the PB level only among parents of children with TD. None of these variables serve as moderating variables among parents of children diagnosed with ASD (See Tables 5 and 6).

**Table 5:** Moderation analyses results among parents of children with ASD (*n* = 101)

Explanatory variable					95% CI	
	B	SE.B	t	*p*	LLCI	ULCI
**Parent ER difficulties as a moderation variable**						
Child behavioral problems (Independent variable)	0.48	0.46	1.04	0.302	− 0.44	1.39
Parent ER difficulties (Moderation variable)	0.52	0.23	2.24*	0.027	0.06	0.98
Child behavioral problems* Parent ER difficulties (Interaction)	− 0.02	0.18	0.13	0.899	− 0.37	0.33
Change in the educational framework (Covariate variable)	0.40	0.19	2.10*	0.039	0.02	0.78
A change in the main caregiver (Covariate variable)	0.79	0.28	2.82**	0.006	0.23	1.35
Change in supports, treatments, or routine (Covariate variable)	0.48	0.19	2.49*	0.015	0.10	0.86
A family/close member injured/killed (Covariate variable)	− 0.51	0.25	2.05	0.043	-1.00	− 0.02
Change in residence following the war (Covariate variable)	− 0.65	0.34	1.92	0.058	-1.32	0.02
**Social support as a moderation variable**						
Child behavioral problems (Independent variable)	− 0.08	0.45	− 0.19	0.851	− 0.97	0.80
Social support (Moderation variable)	− 0.22	0.10	2.18*	0.032	− 0.42	− 0.02
Child behavioral problems* Social support (Interaction)	0.13	0.09	1.48	0.142	− 0.04	0.31
Change in the educational framework (Covariate variable)	0.45	0.20	2.22*	0.029	0.05	0.85
A change in the main caregiver (Covariate variable)	1.19	0.28	4.27***	< 0.001	0.64	1.74
Change in supports, treatments, or routine (Covariate variable)	0.44	0.20	2.21*	0.029	0.05	0.83
A family/close member injured/killed (Covariate variable)	− 0.59	0.26	2.27*	0.026	-1.11	− 0.07
Change in residence following the war (Covariate variable)	− 0.73	0.35	2.05*	0.043	-1.43	− 0.02
**Perceived stress as a moderation variable**						
Child behavioral problems (Independent variable)	0.65	0.66	0.98	0.328	− 0.66	1.95
Perceived stress (Moderation variable)	0.85	0.28	3.04**	0.003	0.29	1.40
Child behavioral problems* perceived stress (Interaction)	− 0.13	0.23	− 0.59	0.558	− 0.58	0.32
Change in the educational framework (Covariate variable)	0.35	0.19	1.87	0.064	− 0.02	0.73
A change in the main caregiver (Covariate variable)	0.88	0.27	3.30**	0.001	0.35	1.42
Change in supports, treatments, or routine (Covariate variable)	0.40	0.19	2.09*	0.040	0.02	0.77
A family/close member injured/killed (Covariate variable)	− 0.53	0.24	2.22*	0.029	-1.01	− 0.06
Change in residence following the war (Covariate variable)	− 0.77	0.33	2.33*	0.022	-1.42	− 0.11

*p <.05, **p <.01, ***p <.001

**Table 6:** Moderation analyses results among parents of children with TD (*n* = 112)

Explanatory variable					95% CI	
	B	SE.B	t	*p*	LLCI	ULCI
**Parent ER difficulties as a moderation variable**						
Child behavioral problems (Independent variable)	− 0.82	0.38	2.12*	0.037	-1.59	− 0.05
Parent ER difficulties (Moderation variable)	0.17	0.13	1.33	0.188	− 0.08	0.42
Child behavioral problems* Parent ER difficulties (Interaction)	0.66	0.17	3.95***	< 0.001	0.33	0.99
Change in residence following the war (Covariate variable)	0.22	0.34	0.655	0.515	− 0.45	0.89
**Social support as a moderation variable**						
Child behavioral problems (Independent variable)	1.65	0.45	3.65***	< 0.001	0.75	2.54
Social support (Moderation variable)	− 0.06	0.06	1.05	0.294	− 0.17	0.05
Child behavioral problems* Social support (Interaction)	− 0.16	0.08	2.17*	0.032	− 0.32	− 0.01
Change in residence following the war (Covariate variable)	0.72	0.35	2.06	0.042	0.03	1.42
**Perceived stress as a moderation variable**						
Child behavioral problems (Independent variable)	-1.53	0.68	2.26*	0.026	-2.88	− 0.19
Perceived stress (Moderation variable)	0.17	0.14	1.19	0.237	− 0.11	0.45
Child behavioral problems* perceived stress (Interaction)	0.85	0.26	3.24**	0.002	0.33	1.37
Change in residence following the war (Covariate variable)	− 0.16	0.42	0.37	0.711	− 0.99	0.68

*p <.05, **p <.01, ***p <.001

**Fig. 1:**
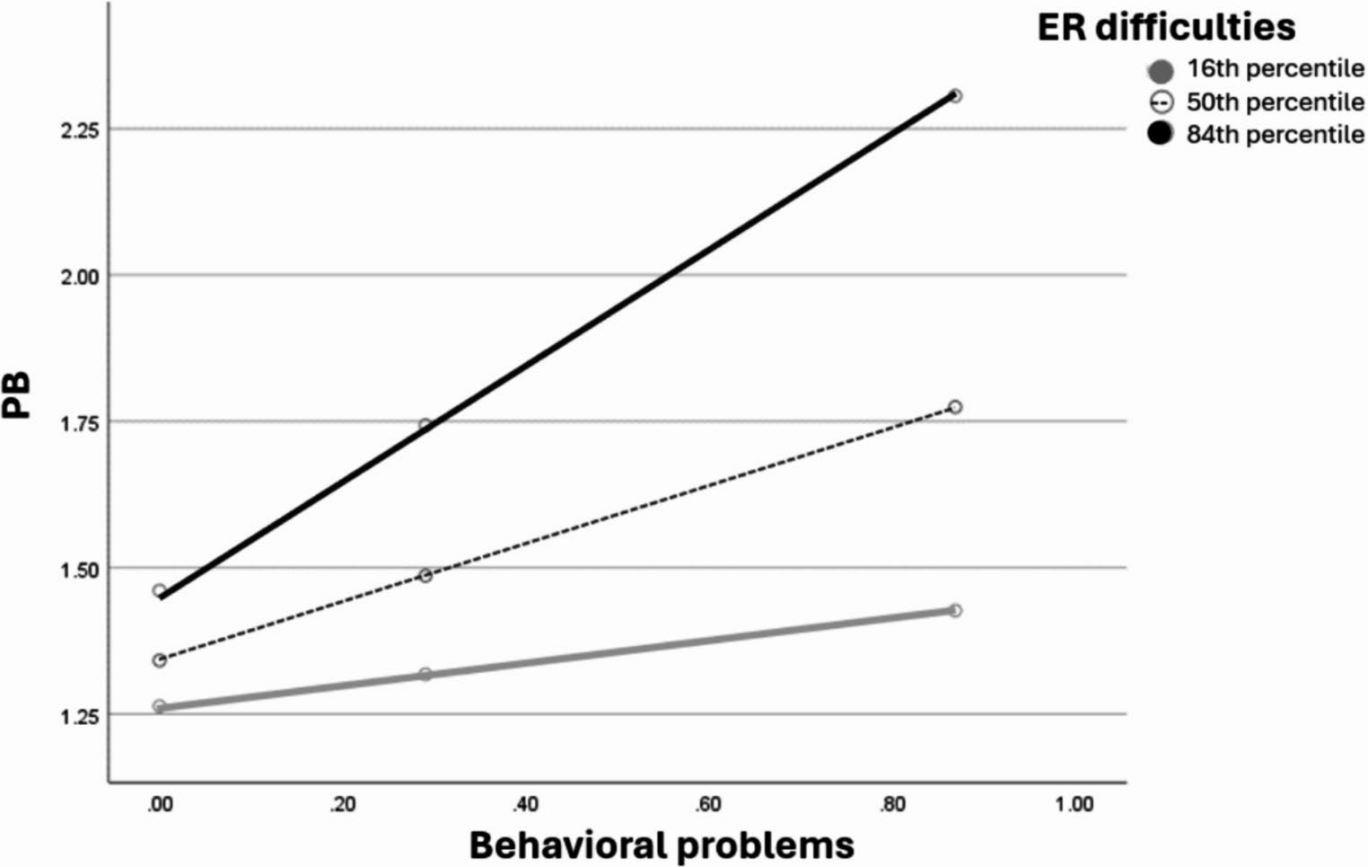
The moderation role of the parent ER difficulties in the direct association between the degree of the child’s behavioral problems and the PB degree during wartime among parents of children with TD presented as percentiles. The percentiles indicate 3 representative values of parent ER difficulties (i.e., low, medium, and high)

**Fig. 2:**
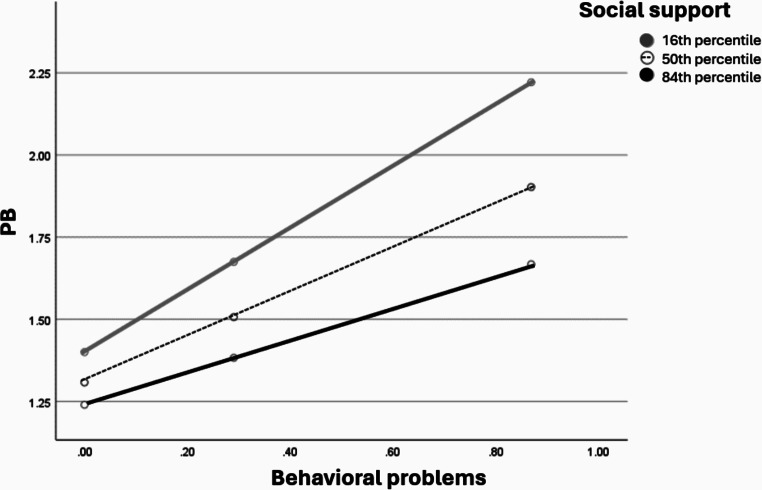
The moderation role of the parent perceived social support in the direct association between the degree of the child’s behavioral problems and the PB degree during wartime among parents of children with TD presented as percentiles. The percentiles indicate 3 representative values of parent perceived social support (i.e., low, medium, and high)

**Fig. 3:**
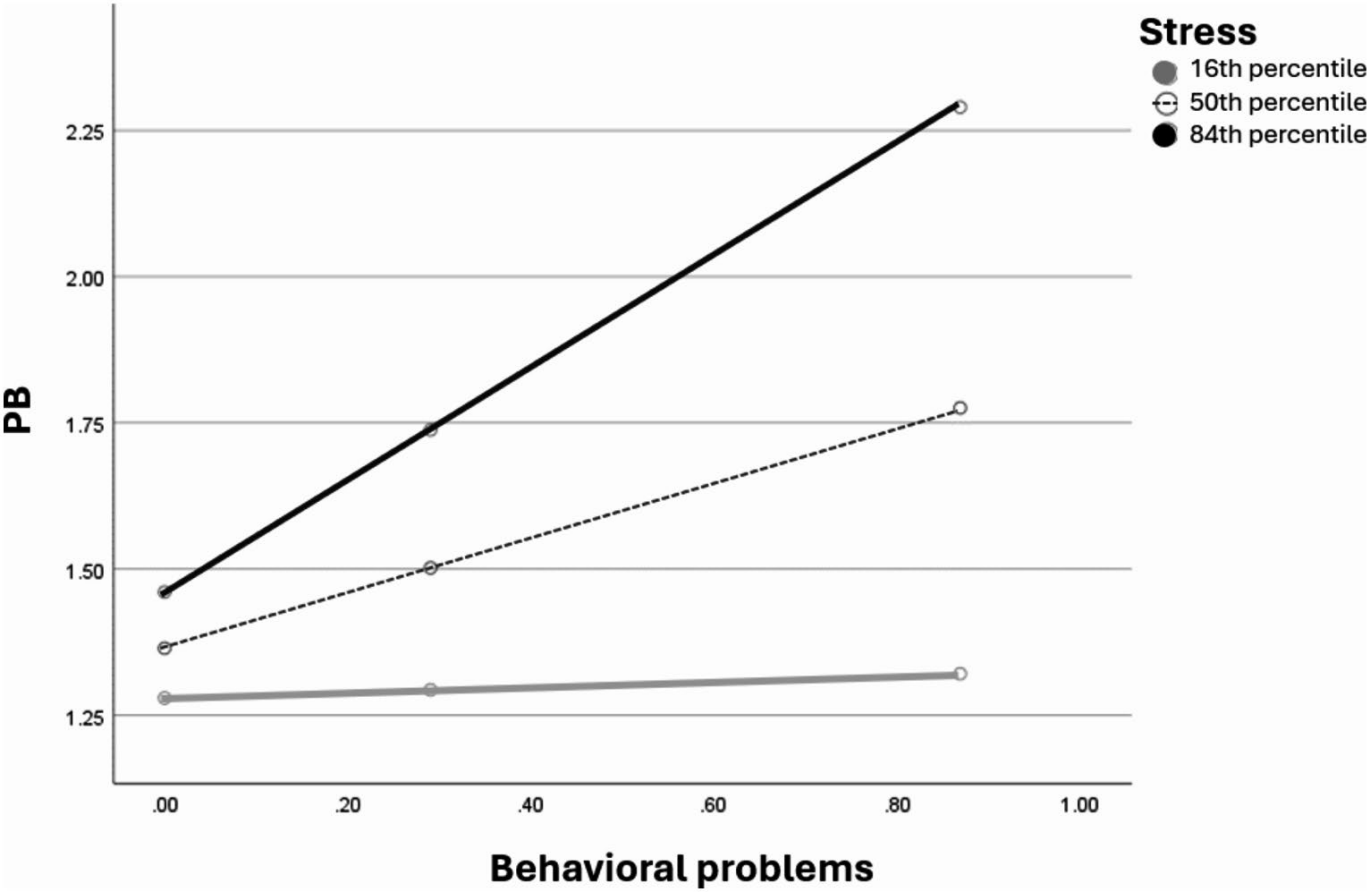
The moderation role of parent stress in the direct association between the degree of the child’s behavioral problems and the PB degree during wartime among parents of children with TD presented as percentiles. The percentiles indicate 3 representative values of parent stress (i.e., low, medium, and high)

Figures 1, 2 and 3 visualizes the results of the moderation role of the parent ER difficulties, perceived social support and stress in the direct association between the degree of the child behavioral problems and the PB degree during wartime among parents of children with TD.

## Discussion

During crises like war, children with ASD and their caregivers face intensified challenges. Disruptions to specialized education, therapy, and psychological support essential for their well-being threaten to worsen their abilities and behavioral issues rooted in structured routines. This, along with added parental responsibilities due to disruptions in caregiving and education, compounded by diminished social support, heightens the emotional burden on already overwhelmed caregivers (Alhuzimi, 2021; Kaba et al., 2023).

In the present study, significant differences were observed in the levels of ER difficulties, stress, perceived social support, child behavioral problems, and PB reported by parents of children diagnosed with ASD compared to those of children with TD during the “Swords of Iron” war. It was also found that the child’s behavioral problems were positively correlated with parental ER difficulties, stress, and PB, and negatively correlated with parental perceived social support. It was also discovered that parents’ ER difficulties, social support, and perceived stress serve as moderating variables between the degree of the child’s behavioral problems and the level of PB only among parents of children with TD, while none of these variables serve as moderating variables among parents of children diagnosed with ASD.

This discussion will address three main issues: (1) The higher levels of ER difficulties, stress, and PB, and the lower level of perceived social support among parents of children diagnosed with ASD during wartime compared to parents of children with TD. (2) The associations between parental ER, social support, and perceived stress with child behavioral problems and PB during wartime. (3) The role parents’ ER, social support, and stress play in the association between child behavioral problems and PB during the “Swords of Iron” war among parents of children with ASD and parents of children with TD.

### Differences in ER Difficulties, Stress, PB and Perceived Social Support During Wartime

Our findings align with existing literature, both during routine periods and amidst the COVID-19 pandemic, indicating that parents of children with ASD typically experience heightened levels of stress (White et al., 2021), ER difficulties (Aydin, 2023), PB (Ardic, 2020), and diminished social support (Kuru & Piyal, 2018). Even amidst the distinct challenges presented by wartime conditions, it seems that parents of children with ASD continue to face greater difficulties compared to parents of TD children, whether during normal times or other crises. These findings underscore that families with children diagnosed with ASD constitute a vulnerable group with distinct needs. This vulnerability arises from an intersectional perspective, which illuminates how various marginalized statuses compound to create new forms of disadvantage (Celeste-Villalvir, 2024). The intersection of being civilians in a war zone and caring for a child with a disability mutually influences the health status and healthcare needs of parents in this group. The high levels of stress, difficulties in accessing primary healthcare, and perceived lack of social support among these parents can be attributed to their intersectional position. They face challenges in accessing healthcare, education, secure housing, and stable living conditions, compounded by the responsibilities of caregiving for a child with ASD, which include economic strain and social stigma leading to isolation. Intersectionality theory helps to understand why these parents experience deficits in these areas and underscores the urgency for systematic changes in community health promotion, public health interventions, and public policies to enhance their health outcomes and quality of life (Masquelier, 2023).

### The Associations Between Parents’ ER Difficulties, Stress and Perceived Social Support with Child Behavioral Problems and PB

Our research revealed positive correlations between child behavioral problems, parental stress, ER difficulties, and PB among both groups. The more severe the reported behavioral problems of the child, the higher the parent’s stress level, indicating greater difficulties in ER and elevated levels of PB. This finding is in line with existing literature on parents of children with ASD, which demonstrates a positive correlation between the behavioral problems of the children and their parents’ ER difficulties. This suggests that when children with ASD exhibit more problematic behaviors, their parents experience increased levels of ER difficulties, and vice versa (Aydin, 2023; Kocak et al., 2023). Similarly, the literature also illustrates a positive correlation between the behavioral problems of children with ASD and parental stress and PB, both in routine settings (Alhuzimi, 2021; Ardic & Olcay, 2021; Argumedes et al., 2018; Shepherd et al., 2018; White et al., 2021) and during emergencies (Bentenuto et al., 2021; Manning et al., 2021; Mutluer et al., 2020; White et al., 2021; Kaba et al., 2023ük et al., 2021).

Furthermore, our study identified a negative correlation between child behavioral problems and parents’ perceived social support, as well as between the level of perceived social support and PB. While the former correlation was evident across both study groups, the latter was observed solely among parents of children with ASD. Social support, particularly the degree of satisfaction derived from it, has emerged as a significant protective factor against PB, transcending personality traits and sociodemographic factors (Ardic, 2020; Lin et al., 2022; Yamoah & Brown, 2023). Our findings corroborate the conclusions drawn from the Meta-analysis conducted by Kocak et al. (2023), which highlighted how the distinct needs and behaviors associated with ASD can impede families’ participation in social activities, leading parents to feel isolated from their social circles, extended family, and broader society, fostering feelings of loneliness and detachment. Moreover, societal stigma and judgment concerning the child’s compulsive behaviors may exacerbate burnout by discouraging parents from seeking assistance or openly discussing their challenges (Lamba et al., 2022; Keville et al., 2021; Kurşun, 2018).

### The Moderating Role of Parents’ ER, Perceived Social Support, and Stress in the Association Between Child Behavioral Problems and PB

Our findings revealed that parents’ ER difficulties, social support, and perceived stress are moderating variables between the degree of the child’s behavioral problems and the level of PB only among parents of children with TD, while none of these variables serve as moderating variables among parents of children diagnosed with ASD. These findings could be elucidated by the Balance between Risks and Resources (BR2) theory proposed by Mikolajczak and Roskam (2018). This theory characterizes PB as a syndrome specific to certain contexts, emerging among parents experiencing chronic stress from parenting. It results from an imbalance between the demands of the parenting role and the resources needed for coping. The BR2 theory emphasizes balance and does not view resources as merely the absence of risks, but as positive forces actively working against them. It addresses risk factors and resources unique to parenting, such as parenting styles and parent-child relationships. The BR2 is not just a theoretical framework, but also a practical tool for diagnosis and treatment through a dedicated measurement tool (BR2) that allows for the assessment of the level of risk and protection for each parent. According to this theory, the role of parenting a child with ASD, coupled with the significant responsibility of addressing developmental and behavioral challenges, may be perceived as an inherent risk factor beyond that of parents raising a child with TD.

Therefore, the scale of risk factors weighs more heavily on the side of parents of children with ASD compared to those of TD children. Consequently, the resources that effectively mitigate risks for parents of typically developing children may not sufficiently counterbalance the heightened risks faced by parents of children with ASD.

A compelling observation supporting this notion of heightened risk among parents of children with ASD is that changes in educational settings, in the main caregiver and in treatments emerged as the significant predictors of PB within this group, whereas relocation was identified as the primary predictor among parents of TD children. The challenges experienced by parents of children with ASD are exacerbated during wartime due to the heightened sensitivity of these children to disruptions in their routines (Yilmaz et al., 2021). In each month during the first three months of the war, approximately 150,000 employees were absent from their workplaces due to reserve duty service (The Chief Economist, 2024). Among them were professionals in the fields of education, health, and welfare who are essential for the continuous care of children with ASD and their families. Disruptions to these educational services, healthcare support, diverse therapies, and tailored services become a critical additional stressor for families with ASD children during the tumult of war. This finding aligns with prior research indicating that during the COVID-19 pandemic, alterations in routine and disruptions to children’s special education precipitated severe behavioral issues in ASD children, heightened parental stress levels, challenges in maintaining mental well-being, and adverse impacts on ER. Consequently, the exacerbation of the imbalance between risks and resources during the “Swords of Iron” war introduces a unique scenario potentially reshaping the anticipated risk/resource equilibrium for both groups, albeit in distinct ways. According to the BR2 theory, while this imbalance is a common occurrence among all burned-out parents, the specific risks and resources vary, resulting in unique burnout experiences, particularly in extreme circumstances like wartime. Individual and group disparities play pivotal roles in the demanding wartime context, potentially amplifying the interplay between risks and resources, thus manifesting distinct roles within each group.

Previous studies in the general population have established an association between child behavioral problems and PB, suggesting that ER may moderate this association (Lin et al., 2022; Prikhidko et al., 2020; Swit & Breen, 2023; Vertsberger et al., 2022). However, our research found this moderation effect only among parents of children with TD. Differences between the two groups in ER difficulties, perceived social support, and child behavioral problems likely contribute to these findings. Parents of children with ASD reported higher ER difficulties, lower perceived social support, and more severe child behavioral problems compared to parents of children with TD. These factors suggest that ER and social support may mitigate PB effectively only under specific conditions or thresholds of child behavioral intensity. The heightened PB observed among parents of children with ASD during wartime could be explained by cumulative risk factors such as increased stress, exacerbated behavioral issues in children, and heightened ER difficulties and lack of social support. These factors likely worsen the imbalance between parental risks and resources, leading to higher levels of PB compared to parents of children with TD. The absence of a moderating effect among the ASD group reinforces previous findings, which have generally highlighted that the behavior and emotions of children with ASD are primary factors impacting parental stress, well-being, and PB (Alhuzimi, 2021; Ardic & Olcay, 2021).

### Limitations and Recommendations for Future Research

The study has limitations that warrant consideration. Firstly, participants were recruited through convenience sampling, which may limit the generalizability of the findings. During the “Swords of Iron” war, some areas in both northern and southern Israel were evacuated. However, this study did not distinguish between mandatory and voluntary evacuation; instead, parents were asked about any changes in their residence due to the war in general. Future research should specifically address the factors that impact those two reasons for relocations, since they likely have a deep impact and relationship to the evacuation experiences of families, including those with ASD. Additionally, future studies should involve larger and more diverse samples from across the country and implement random sampling methods which improve sample representativeness and mitigate biases.

Secondly, the study did not explore the socio-economic status of the parents or the children’s gender and age. The two groups of parents didn’t differ in age which is a good proxy for the children’s age, and they did differ in level of education, which is a possible proxy for socio-economic status. Previous research indicating the potential correlation of socio-economic status with parental burnout (Kuru & Piyal, 2018; Lin et al., 2023). Therefore, future studies should, incorporate an examination of socio-economic factors and additional data on the children.

A key limitation of the study is its cross-sectional design, which prohibits establishing causal relationships. Moreover, the absence of a comparison group of parents of children with ASD and TD during routine times, rather than wartime conditions, hampers the ability to gauge the precise impact of the current war on the variables under investigation. Although Israel residents constantly cope with stress associated with a prolonged state of war, traumas, and emergency routine (Dinur, 2024), the unprecedented damage, challenges and effects of the “Swords of Iron” war may still introduce, alter, or eliminate factors included in BR2 theory, or modify their relative significance. Future studies could employ longitudinal designs to offer a more comprehensive understanding of the relationships between children’s behavioral changes, parental internal and external resources, and PB during wartime. Additionally, the BR2 assessment tool )Roskam et al., 2017) lacks the specific risk factor of having a child with ASD, which is crucial to consider. Future research should explore the overall impact of having a disabled child on the presence, absence, level, or weight of other risk and resource factors by comparing parents of children with and without ASD.

Finally, our study only examined the children’s irritability and hyperactivity/ noncompliance subscales from the ABC, based on previous studies indicating their significant influence on PB and parents’ well-being (Kaat et al., 2014; Kaba et al., 2023). There is disagreement in the literature regarding whether ASD symptoms and stereotypical behaviors exert an additional impact beyond that of behavioral and emotional issues (Kaba et al., 2023tük et al., 2021; Yılmaz et al., 2021). Furthermore, these studies were not conducted during wartime. It is conceivable that the extreme circumstances of wartime have amplified the influence of children’s stereotypical behaviors, potentially resulting in a stronger impact on parents during this specific time. Future research should explore other aspects of behavioral problems and ASD symptoms to provide a more comprehensive understanding.

### Recommendations for Policy

During wartime, access to therapeutic services, online or in-person support groups, and nurturing relationships is vital for maintaining the mental well-being of both parents and children. Financial support and government policies also play significant roles in determining PB levels (Kocak et al., 2023). Addressing burnout-related anxiety necessitates acknowledgment and support through mental health resources, alongside effective coping strategies and family cohesion to manage emotional challenges. Families of individuals with ASD are particularly vulnerable during crises and require specialized services and additional support. Given the significant risk that a child’s behavioral problems pose to parental PB which is a fundamental factor affecting well-being, intervention programs must prioritize the reduction of both the intensity and frequency of these disruptive behaviors. The interaction between a child’s behavior and the parents’ mental state is bidirectional; that is, while a child’s behavior influences parental stress and distress levels, the parents’ emotional state concurrently impacts the child’s behavior. Therefore, it is crucial to address both individual parental well-being and family dynamics when supporting families with children with ASD. To effectively promote the well-being of children with ASD and their parents, holistic intervention approaches are required that address the needs of both parties (Shepherd et al., 2024). These interventions should emphasize enhancing positive communication and interaction between parents and children, developing effective coping strategies, and providing emotional and practical support for both parents and children. Healthcare providers should closely monitor parents of children with ASD and develop resources to enhance coping and reduce stress (Ardic, 2020). Interventions aimed at reducing PB should prioritize high-quality social support over quantity. The literature underscores the pivotal role of social support in aiding parents of children with ASD in managing stress and improving mood (Yilmaz et al., 2021). Comprehensive consideration of parents’ psychological well-being is crucial, especially in minimizing acute stress that may impair effective coping strategies (Prikhidko & Swank, 2019). Tailored interventions for parents of children with ASD should address their specific needs, particularly focusing on behavioral management strategies. Remote interventions adaptable for emergencies are also essential to support children with ASD and their families. Further research is needed to better understand factors contributing to PB reduction in this population, as our study indicates that the variables examined do not effectively moderate PB.

## Data Availability

The data that support the findings of this study are available from the corresponding author, upon reasonable request.

## References

[CR1] Aldao, A., Nolen-Hoeksema, S., & Schweizer, S. (2010). Emotion-regulation strategies across psychopathology: A meta-analytic review. *Clinical Psychology Review*, *30*(2), 217–237. 10.1016/j.cpr.2009.11.004 20015584 10.1016/j.cpr.2009.11.004

[CR2] Alhuzimi, T. (2021). Stress and emotional wellbeing of parents due to change in routine for children with Autism Spectrum disorder (ASD) at home during COVID-19 pandemic in Saudi Arabia. *Research in Developmental Disabilities*, *108*, 103822. 10.1016/j.ridd.2020.103822 33271447 10.1016/j.ridd.2020.103822

[CR3] Aman, M. G., Singh, N. N., Stewart, A. W., & Field, C. (1985). The aberrant behavior checklist: A behavior rating scale for the assessment of treatment effects. *American Journal of Mental Deficiency*, *89*(5), 485–491. 3993694

[CR4] American Psychological Association (2022). Diagnostic and statistical manual of mental disorders fifth edition text revision DSM-5-TR. https://www.appi.org/dsm-5-tr

[CR5] Amorim, R., Catarino, S., Miragaia, P., Ferreras, C., Viana, V., & Guardiano, M. (2020). The impact of COVID-19 on children with an autism spectrum disorder. *Revista De Neurologia*, *71*(8), 285–291. 10.33588/rn.7108.2020381 33034366 10.33588/rn.7108.2020381

[CR6] Ardic, A. (2020). Relationship between parental burnout level and perceived social support levels of parents of children with autism spectrum disorder. *International Journal of Educational Methodology*, *6*(3), 533–543. 10.12973/ijem.6.3.533

[CR7] Ardıc, A., & Olçay, S. (2021). Investigation of the relationship between the burnout level of parents of children with autism spectrum disorder (asd) and asd symptom level and family needs by regression analysis. *Education and Science*, *46*(206), 459–471.

[CR8] Argumedes, M., Lanovaz, M. J., & Larivée, S. (2018). Brief report: Impact of challenging behavior on parenting stress in mothers and fathers of children with autism spectrum disorders. *Journal of Autism and Developmental Disorders*, *48*(7), 2585–2589. 10.1007/s10803-018-3513-1 29478155 10.1007/s10803-018-3513-1

[CR9] Aydin, A. (2023). Examining the Mediating Role of mindful parenting: A study on the relationship between parental emotion regulation difficulties and problem behaviors of children with ASD. *Journal of Autism and Developmental Disorders*, *53*(5), 1873–1883. 10.1007/s10803-022-05455-9. https://doi-org.elib.openu.ac.il/35089435 10.1007/s10803-022-05455-9

[CR10] Bentenuto, A., Mazzoni, N., Giannotti, M., Venuti, P., & De Falco, S. (2021). The psychological impact of Covid-19 pandemic in Italian families of children with neurodevelopmental disorders. *Research in Developmental Disabilities*, *109*, 103840. 10.1016/j.ridd.2020.103840 33383468 10.1016/j.ridd.2020.103840 PMC9186313

[CR11] Bonanno, G. A., & Burton, C. L. (2013). Regulatory flexibility: An individual differences perspective on coping and emotion regulation. *Perspectives on Psychological Science*, *8*(6), 591–612. 10.1177/1745691613504116 26173226 10.1177/1745691613504116

[CR12] Brandão, T., Diniz, E., Basto-Pereira, M., & Babore, A. (2024). Emotion regulation and parental burnout: A systematic review and meta-analysis. *Clinical Psychology: Science and Practice*, *31*(1), 97–109. 10.1037/cps0000181.supphttps://doi-org.elib.openu.ac.il/ (Supplemental).

[CR13] Burisch, M. (2006). *Das burnout-syndrom: Theorie Der Inneren Erschopfung*. Spinger Medizin.

[CR14] Celeste-Villalvir, A., Kovic, C., & Argüelles, F. (2024). The intersectional impact of disability and immigration on Health: A Health needs Assessment of immigrants living with Spinal Cord Injury in Houston, Texas. *Community Health Equity Research & Policy*, *44*(2), 209–218. 10.1177/2752535X221132445. https://doi-org.elib.openu.ac.il/36670517 10.1177/2752535X221132445

[CR15] Ćerimović, E. (2023). At risk and overlooked: Children with disabilities and armed conflict. *International Review of the Red Cross*, *105*(922), 192–216. 10.1017/S181638312200087X. https://doi-org.elib.openu.ac.il/

[CR16] Çi̇men, İ. D., Yeği̇n, Z., Gümüşsoy, A. S., & Kapucu, T. (2023). Children with Special Educational needs and parental burnout during the pandemic Lockdown Period. *Journal of Pediatric Disease / Cocuk Hastaliklari Dergisi*, *17*(6), 466–475. 10.12956/tchd.1317146. https://doi-org.elib.openu.ac.il/

[CR17] Cohen, J. (1987). *Statistical power analysis for the behavioral sciences* (2nd ed.).). Lawrence Erlbaum Associates.

[CR18] Cohen, S., Kamarck, T., & Mermelstein, R. (1983). A Global measure of perceived stress. *Journal of Health and Social Behavior*, *24*(4), 385–396. 10.2307/2136404 6668417

[CR19] Dinur, A. (2024). A stressful identity: Jews, other nations, and other religions. In A. Dinur, I. Ronen, & V. Weiss (Eds.), *Israeli Culture and Emergency Routine: Normalizing stress* (pp. 177–197). Lexington Books.

[CR20] Etikan, I., Musa, S. A., & Alkassim, R. S. (2015). Comparison of convenience sampling and purposive sampling. *American Journal of Theoretical and Applied Statistics*, *5*(1). 10.11648/j.ajtas.20160501.11. Article 1.

[CR21] Findling, Y., Barnoy, S., & Itzhaki, M. (2023). Burden of treatment, emotion work and parental burnout of mothers to children with or without special needs: A pilot study. *Current Psychology: A Journal for Diverse Perspectives on Diverse Psychological Issues*, *42*(22), 19273–19285. 10.1007/s12144-022-03074-2. https://doi-org.elib.openu.ac.il/

[CR22] Gratz, K. L., & Roemer, L. (2004). Multidimensional Assessment of emotion regulation and dysregulation: Development, factor structure, and initial validation of the difficulties in emotion regulation scale. *Journal of Psychopathology and Behavioral Assessment*, *26*(1), 41–54. 10.1023/b:joba.0000007455.08539.94. https://doi-org.elib.openu.ac.il/

[CR23] Gross, J. J. (1998). The emerging field of emotion regulation: An integrative review. *Review of General Psychology*, *2*(3), 271–299. 10.1037/1089-2680.2.3.271

[CR24] Hallion, L. S., Steinman, S. A., Tolin, D. F., & Diefenbach, G. J. (2018). Psychometric properties of the difficulties in emotion regulation scale (DERS) and its short forms in adults with emotional disorders. *Frontiers in Psychology*, *9*, 539. 29725312 10.3389/fpsyg.2018.00539 PMC5917244

[CR25] Hayes, A. F. (2018). Partial, conditional, and moderated mediation: Quantification, inference, and interpretation. *Communication Monographs*, *85*(1), 4–40.

[CR26] Holly, L. E., Buchanan, M., & Bowling, A. R. (2024). Parental burnout and emotion regulation in context: Considerations for science and practice. *Clinical Psychology: Science and Practice*, *31*(1), 113–116. 10.1037/cps0000200. https://doi-org.elib.openu.ac.il/

[CR27] Hsiao, Y. (2016). Pathways to mental health-related quality of life for parents of children with autism spectrum disorder: Roles of parental stress, children’s performance, medical support, and neighbor support. *Research in Autism Spectrum Disorders*, *23*, 122–130.

[CR28] Kaat, A. J., Lecavalier, L., & Aman, M. G. (2014). Validity of the aberrant behavior checklist in children with Autism Spectrum Disorder. *Journal of Autism and Developmental Disorders*, *44*(5), 1103–1116. 10.1007/s10803-013-1970-0. https://doi-org.elib.openu.ac.il/24165702 10.1007/s10803-013-1970-0

[CR29] Kaba, D., Hasanlı, J., Efe, A., Yavuz-Çolak, M., & AkınSarı, B. (2023). Predictors of burnout and distress in parents of children with autism spectrum disorder during COVID-19 home confinement. *Children’s Health Care*, *52*(4), 409–429. 10.1080/02739615.2022.2119974

[CR30] Kerr, M. L., Rasmussen, H. F., Buttitta, K. V., Smiley, P. A., & Borelli, J. L. (2021). Exploring the complexity of mothers’ realtime emotions while caregiving. *Emotion*, *21*(3), 545–556. 10.1037/emo0000719 31916791 10.1037/emo0000719

[CR31] Keville, S., Meek, C., & Ludlow, A. K. (2021). Mothers’ perspectives of co-occurring fatigue in children with autism spectrum disorders. *Fatigue: Biomedicine Health & Behavior*, *9*(4), 209–226. 10.1080/21641846.2021.2008169

[CR32] Koçak, F., Çevik, Ö., & Kızılkaya, H. (2023). Views of parents of a child diagnosed with Autism Spectrum Disorder on Burnout: A Meta-synthesis study. *International Journal of Modern Education Studies*, *7*(2), 435–456. 10.51383/ijonmes.2023.335

[CR33] Kurşun, Z. (2018). Yaygın gelişimsel bozukluğu olan ve normal gelişim gösteren çocukların anne babalarının stres düzeyleri ve stresle başa çıkma yollarının karşılaştırılması (Tez No: 491495) [Comparison of stress levels and ways of coping with stress of parents of children with pervasive developmental disorder and children with normal development (Thesis No:491495)]. Master’s thesis, Istanbul Commerce University. YÖK Thesis Center. https://tez.yok.gov.tr/UlusalTezMerkezi/giris.jsp

[CR34] Kuru, N., & Piyal, B. (2018). Perceived Social Support and Quality of Life of parents of children with autism. *Nigerian Journal of Clinical Practice*, *21*(9), 1182–1189. 10.4103/njcp.njcp_13_18. https://doi-org.elib.openu.ac.il/30156205 10.4103/njcp.njcp_13_18

[CR35] Kütük, M. Ö., Tufan, A. E., Kılıçaslan, F., Güler, G., Çelik, F., Altıntaş, E., et al. (2021). High depression symptoms and burnout levels among parents of children with autism spectrum disorders: A multi-center, cross-sectional, case-control study. *Journal of Autism and Developmental Disorders*, *51*(11), 4086–4099. 10.1007/s10803-021-04874-4 33459915 10.1007/s10803-021-04874-4

[CR36] Lamba, N., Tonder, A. V., Anita Shrivastava, A., & Raghavan, A. (2022). Exploring challenges and support structures of mothers with children with Autism Spectrum Disorder in the United Arab Emirates. *Research in Developmental Disabilities*, *120*, 104138. 10.1016/j.ridd.2021.104138 34864432 10.1016/j.ridd.2021.104138

[CR37] Lin, G. X., Hansotte, L., Szczygieł, D., Meeussen, L., Roskam, I., & Mikolajczak, M. (2021). Parenting with a smile: Display rules, regulatory effort, and parental burnout. *Journal of Social and Personal Relationships*, *38*(9), 2701–2721. 10.1177/02654075211019124

[CR38] Lin, G. X., Goldenberg, A., Arikan, G., Brytek-Matera, A., Czepczor-Bernat, K., Manrique-Millones, D., Mikolajczak, M., Overbye, H., Roskam, I., Szczygieł, D., Ustundag-Budak, A. M., & Gross, J. J. (2022). Reappraisal, social support, and parental burnout. *British Journal of Clinical Psychology*, *61*(4), 1089–1102. 10.1111/bjc.12380. https://doi-org.elib.openu 35852015 10.1111/bjc.12380

[CR39] Lin, Y., Wang, Y., Lin, C., Ni, Q., Jia, R., Chang, Y., & Qi, Y. (2023). The mediating role of perceived social support: Alexithymia and parental burnout in parents of children with autism spectrum disorder. *Frontiers in Psychology*, *14*, 1139618. 10.3389/fpsyg.2023.1139618 37359855 10.3389/fpsyg.2023.1139618 PMC10290202

[CR40] Lincoln, T. M., Schulze, L., & Renneberg, B. (2022). The role of emotion regulation in the characterization, development and treatment of psychopathology. *Nature Reviews Psychology*, *1*(5), 272–286. 10.1038/s44159-022-000404

[CR41] Luther, E. H., Canham, D. L., & Cureton, V. Y. (2005). Coping and social support for parents of children with autism. *The Journal of School Nursing*, *21*(1), 40–47. 15660493 10.1622/1059-8405(2005)021[0040:cassfp]2.0.co;2

[CR42] Manning, J., Billian, J., Matson, J., Allen, C., & Soares, N. (2021). Perceptions of families of individuals with autism spectrum disorder during the COVID-19 crisis. *Journal of Autism and Developmental Disorders*, *51*(8), 2920–2928. 10.1007/s10803-020-04760-5 33090358 10.1007/s10803-020-04760-5 PMC7578441

[CR43] Masquelier, C. (2023). Intersectional socialism: Rethinking the socialist future with intersectionality theory. *Sociology*, *57*(2), 366–381. https://doi-org.elib.openu.ac.il/10.1177/00380385221131143

[CR44] McRae, K., & Gross, J. J. (2020). Emotion regulation. *Emotion*, *20*(1), 1–9. 10.1037/emo0000703 31961170 10.1037/emo0000703

[CR45] Mikolajczak, M., & Roskam, I. (2018). A theoretical and clinical framework for parental burnout: The balance between risks and resources. *Frontiers in Psychology*, *9*, 886. 10.3389/fpsyg.2018.00886 29946278 10.3389/fpsyg.2018.00886 PMC6006266

[CR46] Mikolajczak, M., Gross, J. J., & Roskam, I. (2019). Parental burnout: What is it and why does it matter? *Clinical Psychological Science*, *7*(6), 1319–1329.

[CR47] Mutluer, T., Doenyas, C., & Aslan Genc, H. (2020). Behavioral ımplications of the covid-19 process for autism spectrum disorder, and ındividuals’ comprehension of and reactions to the pandemic conditions. *Frontiers in Psychiatry*, *11*, 561882. 10.3389/fpsyt.2020.561882 33304279 10.3389/fpsyt.2020.561882 PMC7701051

[CR48] Prikhidko, A., & Swank, J. M. (2019). Examining parent anger and emotion regulation in the context of intensive parenting. *The Family Journal*, *27*(4), 366–372. 10.1177/1066480719855371

[CR49] Prikhidko, A., Long, H., & Wheaton, M. G. (2020). The effect of concerns about COVID-19 on anxiety, stress, parental burnout, and emotion regulation: The role of susceptibility to digital emotion contagion. *Frontiers in Public Health*, *8*, 567250. 10.3389/fpubh.2020.567250 33392126 10.3389/fpubh.2020.567250 PMC7775569

[CR50] Rodriguez, M., Bellet, B. W., & McNally, R. J. (2020). Reframing time spent alone. Reappraisal buffers the emotional effects of isolation. *Cognitive Therapy and Research*, *44*(*6*), 1052–1067. 10.1007/s10608-020-10128-x 10.1007/s10608-020-10128-x PMC7335222 32836563

[CR52] Roskam, I., Raes, M. E., & Mikolajczak, M. (2017). Exhausted parents: Development and preliminary validation of the parental burnout inventory. *Frontiers in Psychology*. 10.3389/fpsyg.2017.00163. 8https://doi-org.elib.openu.ac.il/28232811 10.3389/fpsyg.2017.00163 PMC5298986

[CR53] Roskam, I., Brianda, M. E., & Mikolajczak, M. (2018). A Step Forward in the conceptualization and measurement of parental burnout: The parental Burnout Assessment (PBA). *Frontiers in Psychology*. 10.3389/fpsyg.2018.00758. 9https://doi-org.elib.openu.ac.il/29928239 10.3389/fpsyg.2018.00758 PMC5998056

[CR54] Salari, N., Rasoulpoor, S., Rasoulpoor, S., Shohaimi, S., Jafarpour, S., Abdoli, N., Khaledi-Paveh, B., & Mohammadi, M. (2022). The global prevalence of autism spectrum disorder: A comprehensive systematic review and meta-analysis. *Italian Journal of Pediatrics*, *48*(1), 1–16. 10.1186/s13052-022-01310-w. https://doi-org.elib.openu.ac.il/35804408 10.1186/s13052-022-01310-w PMC9270782

[CR55] Santelices, M., Narvaez, S., Escobar, M. J., Oyarce, D., & van Bakel, H. (2022). Burnout and parental emotional regulation strategies: A study in the context of the COVID-19 pandemic in Chile. *Terapia Psicológica*, *40*(2), 153–170. 10.4067/S0718-48082022000200153

[CR56] Shahar, T., & Lerer, M. (2024, July). *Netunim me’udkanim al mefunim mehatzafon umehadarom [Updated data on evacuees from the north and south].* Haknesset, Merkaz HaMechkar VeHaMeida [The Knesset, Center for Research and Information]. https://fs.knesset.gov.il/globaldocs/MMM/bb4ad946-3c2d-ef11-815f-005056aac6c3/2_bb4ad946-3c2d-ef11-815f-005056aac6c3_11_20597.pdf

[CR57] Shepherd, D., Landon, J., Taylor, S., & Goedeke, S. (2018). Coping and care-related stress in parents of a child with autism spectrum disorder. *Anxiety Stress & Coping*, *31*(3), 277–290. 10.1080/10615806.2018.1442614 29463108 10.1080/10615806.2018.1442614

[CR58] Shepherd, D., Buchwald, K., Siegert, R. J., & Vignes, M. (2024). Using network analysis to identify factors influencing the heath-related quality of life of parents caring for an autistic child. *Research in Developmental Disabilities*, *152*, 104808. 10.1016/j.ridd.2024.104808. https://doi-org.elib.openu.ac.il/39067240 10.1016/j.ridd.2024.104808

[CR59] Sörman, K., Garke, M. Å., Isacsson, N. H., Jangard, S., Bjureberg, J., Hellner, C., Sinha, R., & Jayaram, L. N. (2022). Measures of emotion regulation: Convergence and psychometric properties of the difficulties in emotion regulation scale and emotion regulation questionnaire. *Journal of Clinical Psychology*, *78*(2), 201–217. 10.1002/jclp.23206. https://doi-org.elib.openu.ac.il/34217149 10.1002/jclp.23206

[CR60] Swit, C. S., & Breen, R. (2023). Parenting during a pandemic: Predictors of parental burnout. *Journal of Family Issues*, *44*(7), 1817–1837. 10.1177/0192513X211064858

[CR61] Taub Center researchers. (2023, December). The October War and Its Impact on Israel’s Society and Economy. *State of the Nation Report: Society, Economy and Policy 2023*. Taub Center for Social Policy Studies in Israel.

[CR62] *Ma’afyenei HaNe’edarim Me’avodatam Ketotza’a MiSherut Milu’im BeHodesh December 2023 [Characteristics of Those Absent from Work Due to Reserve Duty Service in December 2023]*. Ministry of The Chief Economist, & Finance (2024, February). https://www.gov.il/BlobFolder/dynamiccollectorresultitem/periodic-review-05022024/he/weekly_economic_review_periodic-review-05022024.pdf

[CR63] Türkoğlu, S., Uçar, H. N., Çetin, F. H., Güler, H. A., & Tezcan, M. E. (2021). The relationship between irritability and autism symptoms in children with ASD in COVID-19 home confinement period. *International Journal of Clinical Practice*, *75*(11), e14742. 10.1111/ijcp.14742 34423522 10.1111/ijcp.14742 PMC8646706

[CR64] Vertsberger, D., Roskam, I., Talmon, A., Van Bakel, H., Hall, R., Mikolajczak, M., & Gross, J. J. (2022). Emotion regulation during the COVID-19 pandemic: Risk and resilience factors for parental burnout (IIPB). *Cognition and Emotion*, *36*(1), 100–105. 10.1080/02699931.2021.2005544 34821543 10.1080/02699931.2021.2005544

[CR65] Wang, K., Goldenberg, A., Dorison, C. A., Miller, J. K., Uusberg, A., Lerner, J. S., et al. (2021). A multi-country test of brief reappraisal interventions on emotions during the COVID-19 pandemic. *Nature Human Behaviour*, *5*(8), 1089–1110. 10.1038/s41562-021-01173-x 34341554 10.1038/s41562-021-01173-x PMC8742248

[CR66] White, L. C., Law, J. K., Daniels, A. M., Toroney, J., Vernoia, B., Xiao, S., Feliciano, P., & Chung, W. K. (2021). Brief report: Impact of COVID-19 on individuals with ASD and their caregivers: A perspective from the SPARK cohort. *Journal of Autism and Developmental Disorders*, *51*(10), 3766–3773. 10.1007/s10803-020-04816-6 33387233 10.1007/s10803-020-04816-6 PMC7775834

[CR67] World Health Organization. (2022). *Autism*. World Health Organization.

[CR68] Yamoah, J., & Brown, L. (2023). Understanding the types of social support that can mitigate parental burnout in mothers of children with medical complexity. *Child: Care Health and Development*, *49*(4), 732–739. 10.1111/cch.13087 36460012 10.1111/cch.13087

[CR69] Yang, B., Chen, B. B., Qu, Y., & Zhu, Y. (2021). Impacts of parental burnout on Chinese youth’s mental health: The role of parents’ autonomy support and emotion regulation. *Journal of Youth and Adolescence*, *50*, 1679–1692. 10.1007/s10964-021-01450-y 34106359 10.1007/s10964-021-01450-y PMC8188764

[CR70] Yılmaz, B., Azak, M., & Şahin, N. (2021). Mental health of parents of children with autism spectrum disorder during COVID-19 pandemic: A systematic review. *World Journal of Psychiatry*, *11*(7), 388. 34327131 10.5498/wjp.v11.i7.388 PMC8311509

[CR71] Zimet, G. D., Dahlem, N. W., Zimet, S. G., & Farley, G. K. (1988). The Multidimensional Scale of Perceived Social Support. *Journal of Personality Assessment*, *52*(1), 30–41. 10.1207/s15327752jpa5201_2 10.1080/00223891.1990.9674095 2280326

